# Bereavement and mental health factors associated with seeking and receiving support following loss among Canadian bereaved adults

**DOI:** 10.1007/s00127-025-03020-0

**Published:** 2025-12-02

**Authors:** Enya Redican, Andy Langford, Philip Hyland, Thanos Karatzias, Mark Shevlin

**Affiliations:** 1 School of Psychology, Ulster University, BT52 1SA, Northern Ireland, Coleraine, Cromore Road UK; 2 Cruse Bereavement Support, One Victoria Villas, TW9 2GW Richmond, UK; 3 Department of Psychology, Maynooth University, Kildare, Ireland; 4 School of Health & Social Care, Edinburgh Napier University, Edinburgh, UK

**Keywords:** Bereavement, Social support, Support sources, Mental health

## Abstract

**Purpose:**

The death of a loved one is one of life’s most ubiquitous events that can increase risk of mental health difficulties. Bereavement support is one of the few factors influencing grief-related mental health outcomes that can be modified after bereavement. This study sought to determine the proportion of bereaved people that want and receive support from different sources following a bereavement, and the bereavement and mental health-related factors associated with wanting and receiving bereavement support.

**Methods:**

Data was derived from a cross-sectional survey of bereaved adults (*n* = 1170) living in Ontario, Canada.

**Results:**

Over a third of the sample (38.9%; *n* = 455) reported wanting support in coping with their loss. These individuals exhibited distinct loss-related characteristics and reported higher levels of anxiety, depression, and prolonged grief symptoms. Most of these individuals received support and a small number of participants who didn’t want support received it regardless. The most common sources of support were family members, friends, and other bereaved individuals, and these sources were generally found to be helpful. Those who accessed multiple types of support were those with the highest levels of anxiety, depression, and prolonged grief symptoms.

**Conclusion:**

Most individuals wanting support after a loss can access it and find it beneficial. The desire for support is closely tied to psychological distress, highlighting the need to prioritize formal support for those in distress and rely on existing social networks for those who are not. Such an approach embodies an ‘assets-based’ bereavement model, enhancing community capacity for effective support provision.

## Introduction

 The death of a loved one is one of life’s most ubiquitous yet emotionally distressing events. While it is common for bereaved individuals to experience intense grief symptoms initially, these symptoms generally subside as individuals come to terms with the finality of their loss and assimilate its reality into their lives [1]. However, for a minority of bereaved individuals, grief remains persistent and debilitating [2–4]. These individuals are more likely to experience pathological grief reactions, such as Prolonged grief Disorder (PGD), which is included as a grief-specific disorder in the 11th version of the International Classification of Diseases (ICD-11; World Health Organisation [WHO], 5]. PGD is characterized by prolonged longing and/or preoccupation with the deceased, along with associated problems like sadness, guilt, anger, and difficulty accepting the death [5]. Recent research shows that the average PGD prevalence is 13%, with rates varying from 5% in probability samples to 16% in non-probability samples [6]. Along with sampling differences, these rates vary depending on the measure used to assess PGD, the nature of the sample, and the cultural and loss-related characteristics of the sample [6]. Other mental health disorders, such as anxiety and depression can also develop following bereavement and affect 10% to 20% of bereaved individuals [7], often co-occurring with PGD [8].

A growing body of evidence identifies risk factors for poor mental health following bereavement, including recency of loss, unexpected or violent deaths, and the death of a child or partner [e.g., 9, 10]. However, all of these are non-modifiable risk factors, and thus, modifiable risk factors that can reduce distress and improve coping abilities have been the focus of considerable research in recent years [11, 12]. One such modifiable factor is bereavement support, which has been shown to significantly influence grief reactions [13, 14] and protect against the negative psychological and physiological effects of loss [15]. Support may be informal, arising from one’s personal network of family, friends, and community members, or formal, provided by professionals or trained volunteers, such as clergy, bereavement support services, and peer groups of the bereaved, as well as clinical sources including family doctors and grief counsellors [17]. Just as there is variation in how individuals respond to the death of a loved one, there are also notable differences in who seeks and receives help, the sources of support, the perceived benefit of that support, and the amount of support received [16–19]. The public health model to bereavement care acknowledges that informal support from family and friends is sufficient for most people, but that individuals at risk of experiencing poor grief outcomes —such as anxiety, depression, or PGD—often need additional, formal support or therapeutic intervention [16]. This is reflected in research showing that most bereaved individuals primarily rely on informal support systems, while professional supports such as bereavement support groups and psychologists are utilized the least [17] and generally used by those at high risk of meeting diagnostic criteria for PGD [18]. Moreover, there are some people, approximately 8–10%, who do not want bereavement support [19]. Research also shows how perceptions of helpfulness (operationalised in previous research as very/quite helpful or a little/not helpful) also vary significantly across different sources of support. Informal support has been shown as being regarded as most helpful by bereaved individuals and formal supports having the highest rates of perceived unhelpfulness, and individuals with greater prolonged grief (PG) symptom severity typically report the highest levels of dissatisfaction with support [18, 19]. Finally, quantity of bereavement support matters; for some bereaved individuals, excessive informal support may feel overwhelming and be perceived as a burden rather than helpful [20]. Notably, while previous studies have examined who seeks and receives support, the sources and perceived benefits of that support, and the amount of support received — and one study has explored this in the context of PG symptoms [18] — no research, to our knowledge, has investigated it across a broad range of loss-related and mental health characteristics. Addressing this gap could provide valuable insights into help-seeking behaviours among bereaved individuals, how effectively current support systems meet the psychological needs of bereaved individuals and who is most likely to benefit from formal supports.

The aim of this study was to provide new and important information on bereavement support and help-seeking, and how these relate to loss-related and mental health characteristics. Specifically, we aimed to (i) describe the loss-related and mental health characteristics of the sample, (ii) determine the proportion of bereaved individuals who want support in coping with their loss, and those who do not, alongside their loss-related characteristics and levels of anxiety, depression, and PGD; (iii) determine the proportion of bereaved individuals who received support and wanted support, those who didn’t receive support and wanted support, and those who received support but did not want it, and explore differences based on loss-related factors; (iv) identify the sources of support used by bereaved individuals and their perceived helpfulness; and (v) examine the association between total number of support types and levels of anxiety, depression, and PGD.

## Methods

### Participants and procedures

The sample for this study was drawn from a larger dataset comprising a representative sample of adults (*N* = 1,310) living in Ontario, Canada. Qualtrics recruited participants via managed, double opt-in research panels using email, SMS, and in-app notifications. Non-probability quota sampling ensured representation by age, gender, and income (benchmarked against Statistics Canada, 2021). Inclusion criteria were that participants were 18 years or older, were residing in Ontario, and could complete the survey in English. Participants included only those who answered “Yes” to “*During your life*,* has someone close to you died (for example*,* a partner*,* parent*,* child*,* close friend)?*”. The final analytic included 1170 participants (89.3%). The mean age of the sample was 48.23 years (SD = 16.65, range = 18–91) and 54.5% (*n* = 638) were female. Over half were in a committed relationship (58.2%, *n* = 681), had a college degree or higher (62.5%, *n* = 733), and were employed (54.7%, *n* = 631). More than two thirds reported living in an urban area (68.4%; *n* = 800) and being born in Canada (76.2%; *n* = 891). Ethical approval was granted from the research ethics committee at Maynooth University, Ireland (approval number: SRESC-2023–36746) and informed consent was provided electronically by all participants.

### Measures

#### Support

Participants were first asked since experiencing their loss, had they (1) ever wanted support in coping with their loss and (2) received any support (from a family member, friend, doctor, therapist) in coping with their loss. Both questions had ‘yes’ or ‘no’ responses. If participants answered ‘yes’ to the second question about receiving support in coping with their loss, they were asked to indicate whether they received help from any of eight listed sources (family members, friends, members of the local community, family doctor, grief counsellor, support services for bereavement, other bereaved people, clergy person) using the following response options: ‘no’, ‘yes, and found it helpful’, or ‘yes, but did not find it helpful’. Participants could indicate multiple sources of support.

#### PG symptoms

The International Grief Questionnaire [IGQ; 21] is a self-report measure of ICD-11 PGD. Participants were instructed to indicate how bothered they have been by each of five symptoms in the last week using a five-point Likert scale with responses ranging from 0 (‘Not at all’) to 4 (‘Extremely’). The IGQ was used to generate severity scores by summing the symptom items. Possible scores range from 0 to 20, with higher scores indicating greater symptom severity. The internal reliability of the IGQ scores in the current study was good (α = 0.86).

#### Generalised anxiety

The International Anxiety Questionnaire [IAQ; 22] is an eight-item self-report measure which assesses generalised anxiety disorder (GAD) as described in the ICD-11. Participants were asked to indicate how often they had been bothered by each of the symptoms over the last several months using a five-point Likert scale with responses ranging from 0 (‘Never’) to 4 (‘Every day’). Possible scores range from 0 to 32, with higher scores indicating greater symptom severity. The internal reliability of the IAQ in the current study was excellent (α = 0.95).

#### Depression

The International Depression Questionnaire [IDQ; 22] is a nine-item self-report measures which assesses depressive episode as defined by ICD-11. Participants were asked to indicate how often they had been bothered by each of the symptoms over the last two weeks using a five-point Likert scale with responses ranging from 0 (‘Never’) to 4 (‘Every day’). Possible scores range from 0 to 36, with higher scores indicating greater symptom severity. The internal reliability of the IDQ in the current study was excellent (α = 0.95).

#### Loss-related variables

Loss-related variables included time since bereavement (within the last six months, six months to a year ago, 1–2 years ago, 2–3 years ago, 3–5 years ago, 6–10 years ago, and more than 10 years ago), nature of the death (anticipated natural death, unexpected natural death, sudden unnatural death, suicide, other), and frequency of contact with the deceased in the year prior to their death (1 = Every day, 2 = Almost every day, 3 = Several times a week, 4 = Several times a month, 5 = A few times in the year, 6 = Not at all during that year). Additionally, participants were provided with a list of different bereavements that can occur during the lifetime and were asked to select those which they had experienced. From this list, participants were then asked to indicate the bereavement that they were most impacted by, with responses categorised as child, partner/spouse, parent, brother or sister, extended family, and friends.

## Analytic approach

Analyses were conducted in two phases. In phase 1, descriptive statistics were reported for all mental health and loss-related variables. Binary logistic regression analyses was then conducted to examine associations between the loss-related variables and wanting support (reference group is not wanting support), with results reported as odds ratios (ORs) with 95% confidence intervals. Independent samples t-tests compared means levels of anxiety, depression, and PG symptoms between those wanting support and those not. The strength of these associations was quantified using Cohen’s [23] were interpreted according to guidelines where values less than 0.30 indicate a small effect, 0.30–0.50 indicate a medium effect, and greater than 0.50 indicate a large effect. Concordance between the proportion who wanted and received support was examined using a chi-square test of independence, and the kappa statistic measured the agreement between these two variables. Agreement levels were interpreted following Cohen’s [23] guidelines: 0–0.20 = no agreement, 0.21–0.39 = minimal agreement, 0.40–0.59 = weak agreement, 0.60–0.79 = moderate agreement, 0.80–0.90 = strong agreement, >0.90 = almost perfect.

Analyses in phase 2 focused on those who received support (*n* = 586). Descriptive statistics were calculated, and binary logistic regression analyses was conducted to examine associations between loss-related variables and wanting support and receiving it (reference group is not wanting support but receiving it). Independent samples t-tests compared means levels of anxiety, depression, and PG symptoms between those wanting support and those not. The strength of these associations was quantified using Cohen’s [24] *d* (< 0.40 = small, 0.40–0.80 = moderate, >0.80 = large). Next, the proportion who received support from the eight sources, and who found these supports helpful was estimated. Finally, a variable representing the total number of supports types received (1, 2, 3, and ≥ 4 supports) was computed and one-way analyses of variance (ANOVAs) were conducted to assess statistically significant differences in mean levels of anxiety, depression, and PG symptoms across the different support levels.

## Results

### Descriptive statistics for mental health and loss-related characteristics

For the overall sample, average depression scores were 10.69 (SD = 12.24; Range = 0–36), average anxiety scores were 12.50 (SD = 9.41; Range = 0–32) and average PG symptoms were 8.28 (SD = 6.11; Range = 0–20). Regarding loss-related characteristics, 5.0% (*n* = 58) experienced a loss within the last six months, 3.9% (*n* = 46) six months to one year ago, 6.5% (*n* = 76) one to two years ago, 8.0% (*n* = 94) two to three years ago, 21.5% (*n* = 251) three to five years ago, 14.5% (*n* = 170) six to ten years ago, and 40.6% (*n* = 475) more than 10 years ago. Nearly half of the bereavements (47.8%; *n* = 559) resulted from anticipated natural deaths, followed by unexpected natural deaths (33.9%; *n* = 397), sudden unnatural deaths (8.7%; *n* = 102), suicide (4.6%; *n* = 54), and other causes (5.0%; *n* = 58). In terms of relationship to the deceased, participants were most commonly affected by the death of a parent (42.4%; *n* = 496), followed by extended family members (30.7%; *n* = 359), friends, acquaintances, or colleagues (9.6%; *n* = 112), siblings (7.1%; *n* = 83), partners/spouses (6.1%; *n* = 71), and children (4.2%; *n* = 49). Finally, regarding contact prior to death, almost a third of participants (28.8%; *n* = 337) reported daily contact with the deceased, while only 3.8% (*n* = 44) had no contact in the year preceding the death.

## Mental health and loss-related characteristics associated with wanting support

More than a third of the sample (38.9%; *n* = 455) reported wanting bereavement support. Compared to not wanting support (*n* = 714), wanting support was significantly associated with time since bereavement (*χ*^*2*^ [6] = 23.93, *p* < 0.001, V = 0.14), nature of death (χ2 [4] = 25.34, *p* < 0.001, V = 0.16), frequency of contact with the deceased (*χ*^*2*^ [4] = 25.34, *p* < 0.001, V = 0.15), and death most affected by (*χ*^*2*^ [5] = 29.91, *p* < 0.001, V = 0.15). The largest proportion wanting support (Table 1) were those bereaved less than six months, grieving a death by suicide, in daily contact with the deceased, and most affected by the death of a partner/spouse. The smallest proportion were those bereaved over 10 years, reporting anticipated natural deaths, no contact at all in the year prior, and those most affected by the death of a parent. Table 1 also shows unadjusted associations between loss-related variables and wanting support. Significant positive predictors include bereavement occurring less than six months ago, 1–2 years, and 2–3 years (vs. >10 years), unexpected natural death, sudden unnatural death, death from suicide, or other (vs. anticipated natural death), being in contact with the deceased every day or almost every day (vs. several times a month) and being most affected by the death of a child or partner/spouse (vs. a parent). As demonstrated in Table 2, average levels of anxiety, depression, and prolonged grief were significantly higher for those who wanted support compared to those who didn’t.

**Table 1:** Loss-related factors associated with wanting bereavement support for the overall sample (*n* = 1169)

Time since bereavement	%	OR	CI
Within the last 6 months	55.2%	**2.54**	(1.46, 4.41)
6 months to a year ago	45.7%	1.73	(0.94, 3.20)
1–2 years	44.7%	**1.67**	(1.02, 2.73)
2–3 years	52.7%	**2.30**	(1.47, 3.61)
3–5 years	39.8%	1.37	(0.99, 1.88)
6–10 years	37.6%	1.25	(0.87, 1.80)
More than 10 years	32.6%	-	-
***Nature of death***			
Anticipated natural death	32.0%	-	-
Unexpected natural death	42.6%	**1.57**	(1.21, 2.06)
Sudden unnatural death	51.5%	**2.25**	(1.47, 3.46)
Death from suicide	51.9%	**2.29**	(1.30, 4.01)
Other	46.6%	**1.85**	(1.07, 3.19)
***Frequency of contact***			
Every day	51.9%	**2.48**	(1.73, 3.55)
Almost every day	40.9%	**1.58**	(1.05, 2.39)
Several times a week	36.7%	1.33	(0.89, 1.99)
Several times a month	30.4%	-	-
A few times in the year	24.0%	0.72	(0.45, 1.15)
Not at all during the year	29.6%	0.96	(0.47, 1.96)
***Death most affected by***			
Child	59.2%	**2.78**	(1.53, 5.06)
Partner or spouse	62.0%	**3.13**	(1.87, 5.22)
Parent	34.3%	-	-
Brother or sister	42.2%	1.40	(0.87, 2.25)
Extended family	38.4%	1.20	(0.90, 1.59)
Friends, acquaintances, or colleagues	35.1%	1.04	(0.68, 1.60)

**Table 2:** Mental health factors associated with wanting bereavement support for the overall sample (*n* = 1169)

	Support	*n*	Mean(sd)	t	Cohen’s d
Depression	Wanted Support	455	16.93 (10.35)	12.64***	0.76
	Didn’t want support	714	9.24 (9.79)		
Anxiety	Wanted Support	455	16.72 (8.99)	−13.18***	0.79
	Didn’t want support	714	9.79 (8.62)		
Prolonged Grief	Wanted Support	455	11.10 (6.04)	−13.27***	0.82
	Didn’t want support	714	6.47 (5.43)		

### Mental health and loss-related characteristics associated with wanting and receiving support

Approximately half of the sample (50.1%; *n* = 586) reported receiving support. Wanting support was significantly associated with receiving support (*χ*^*2*^ [6] = 170.75, *p* < 0.001), with more participants than expected wanting and receiving support (adjusted standardized residual = 13.1). However, the kappa statistic (k = 0.37) indicated only fair agreement, likely due to the 42.5% (*n* = 249) of participants who received support but did not want it. The remainder of analyses were restricted to those wanting and receiving support (*n* = 337) compared to those who received support but did not want it (*n* = 249). Wanting and receiving support was significantly associated with nature of death (*χ*^*2*^ [4] = 18.99, *p* < 0.001, V = 0.18), frequency of contact with the deceased (*χ*^*2*^ [5] = 22.71, *p* < 0.001, V = 0.20), and death most affected by (*χ*^*2*^ [5] = 19.12, *p* = 0.002, V = 0.18), but not time since bereavement (*χ*^*2*^ [6] = 8.18, *p* = 0.225). Table 3 shows the largest proportion wanting and receiving support were those bereaved 2–3 years, grieving a death by suicide, in daily contact with the deceased, and most affected by the death of a child. The smallest proportion were bereaved over 10 years, reporting an anticipated natural death, in contact a few times in the year, and most affected by death of extended family. Table 3 provides unadjusted associations between loss-related variables and membership of the wanting support and receiving it group. Significant positive predictors included unexpected natural and sudden unnatural death (vs. anticipated natural), being in contact with the deceased every day (vs. several times a month) and being most affected by the death of a child, partner/spouse, and brother/sister (vs. a parent). As demonstrated in Table 4 and 5 average levels of anxiety, depression, and PG symptoms were significantly higher for those who received support compared to those who didn’t.

**Table 3:** Loss-related characteristics of those who wanted support and received it (*n* = 586)

Time since bereavement	%	OR	CI
Within the last 6 months	63.9%	1.69	(0.82, 3.51)
6 months to a year ago	66.7%	1.91	(0.74, 4.92)
1–2 years	60.5%	1.47	(0.73, 2.96)
2–3 years	68.4%	**2.07**	(1.12, 3.84)
3–5 years	59.2%	1.39	(0.90, 2.15)
6–10 years	58.2%	1.33	(0.79, 2.24)
More than 10 years	51.1%	-	-
***Nature of death***			
Anticipated natural death	48.9%	-	-
Unexpected natural death	62.4%	**1.74**	(1.20, 2.51)
Sudden unnatural death	62.3%	1.73	(0.94, 3.17)
Death from suicide	82.8%	**5.03**	(1.86, 13.57)
Other	65.6%	2.00	(0.93, 4.31)
***Frequency of contact***			
Every day	69.7%	**2.66**	(1.61, 4.40)
Almost every day	55.0%	1.41	(0.81, 2.47)
Several times a week	53.5%	1.33	(0.77, 2.29)
Several times a month	46.4%	-	-
A few times in the year	43.5%	0.89	(0.47, 1.69)
Not at all during the year	54.5%	1.39	(0.40, 4.85)
***Death most affected by***			
Child	77.4%	**2.94**	(1.22, 7.07)
Partner or spouse	74.4%	**2.49**	(1.20, 5.17)
Parent	53.9%	-	-
Brother or sister	76.5%	**2.78**	(1.21, 6.39)
Extended family	51.4%	0.91	(0.62, 1.33)
Friends, acquaintances, or colleagues	57.4%	1.15	(0.64, 2.09)

**Table 4:** Mental health factors associated with wanting and receiving bereavement support (*n* = 586)

		*n*	Mean (sd)	t	Cohen’s d
Depression	Wanted and Received Support	337	16.09 (10.42)	−7.45***	0.61
	Didn’t want but received support	249	10.09 (9.00)		
Anxiety	Wanted and Received Support	337	16.33 (9.05)	−7.64**	0.64
	Didn’t want but received support	249	10.82 (8.05)		
Prolonged Grief	Wanted and Received Support	337	11.19 (6.13)	−9.19***	0.75
	Didn’t want but received support	249	6.91 (5.12)		

**Table 5:** Modalities of support and their helpfulness among those who received support (*n* = 586)

	Yes	Found it helpful	Didn’t find it helpful
Family members	520 (88.7%)	448 (86.2%)	72 (13.8%)
Friends	504 (86.0%)	435 (86.3%)	69 (13.7%)
Members of the local community	221 (37.7%)	173 (78.3%)	48 (21.7%)
Family doctor	230 (39.2%)	174 (75.7%)	56 (24.3%)
Grief counsellor	181 (30.9%)	143 (79.0%)	38 (21.0%)
Support services for bereavement	156 (26.6%)	125 (80.1%)	31 (19.9%)
Other bereaved people	285 (48.6%)	241 (84.6%)	44 (15.4%)
Clergy person	154 (26.2%)	125 (81.2%)	29 (18.8%)

## Mental health characteristics associated with number of support types

**Fig. 1:**
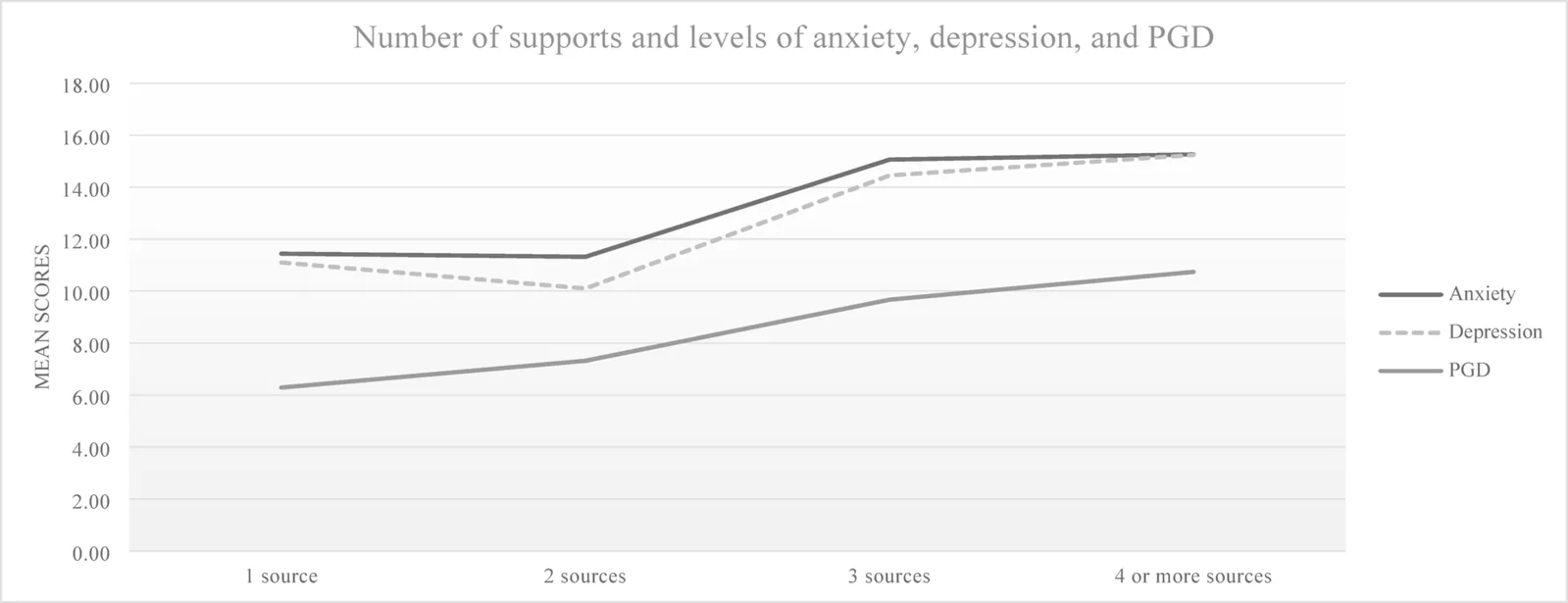
Mean anxiety, depression, and PG symptoms scores according to number of support types

Table 4 shows, for those who received support (*n* = 586), the sources from which they received support and whether they found these supports helpful. Results demonstrates that family members, friends and other bereaved people were the most common sources of support, while clergy, bereavement support services, and grief counsellors were the least common. Regarding their degree of helpfulness, support from friends and family were deemed most helpful and support from the family doctor and members of the local community were deemed the least helpful. The median number of types of support received was 3.00 (M = 3.84, SD = 2.06, Range = 0–8). 8.2% (*n* = 48) reported having one source of support (2 sources = 23.7% (*n* = 139), 3 sources = 19.8% (*n* = 116), 4 or more sources = 48% (*n* = 282)). One-way ANOVAs demonstrated significant associations between number of support types and levels of anxiety [F(3, 581) = 7.96, *p* < 0.001, η2 = 0.04], depression [F(3, 581) = 9.35, *p* < 0.001, η2 = 0.05], and PG symptoms [F(3, 581) = 15.23, *p* < 0.001, η2 = 0.07]. Figure 1 illustrates that mean anxiety, depression and PG symptoms scores were highest for those reporting four or more sources (anxiety mean = 15.26; depression mean = 15.24; PG symptoms mean = 10.74). Mean anxiety (11.32) and depression (10.10) scores were lowest for those reporting two sources, and mean PG symptom scores (6.29) were lowest for those reporting one source. For anxiety, depression, and PGD, scores were significantly lower for those with two sources compared to three or four sources; and for anxiety and PGD, scores were significantly lower for those reporting one source compared to three or four.

## Discussion

This study sought to determine levels of psychological distress among a bereaved population and determine the proportion of bereaved people who wanted and received support from different sources following a bereavement, and the bereavement and mental health-related factors associated with wanting and receiving bereavement support. Findings demonstrated that levels of PG symptoms (Mean = 8.28) were slightly higher in the present study compared to those observed in prior studies conducted in the UK (Mean = 6.30) and Ireland (Mean = 5.10) using similar measures [21], while levels of depression and anxiety were largely similar to prior studies [25]. The reasons for the higher levels of PG symptoms in the present study are unclear given similar demographic and loss-related characteristics of the samples, and thus, further research is required to explore whether particular social or cultural factors may be driving the higher levels of PG symptoms in the present sample. Findings also revealed that just over one-third wanted support in coping with their loss, particularly those who experienced a recent bereavement, had regular contact with the deceased prior to their death, experienced an unnatural, unexpected, or violent death, and were affected by the loss of a child and partner or spouse. These are widely recognized as risk factors for pathological grief reactions [e.g., 10], explaining why individuals who wanted support also exhibited higher levels of anxiety, depression, and PGD. As expected, participants who expressed a desire for support were more likely to receive it, suggesting that the factors contributing to vulnerability to pathological grief may also play a role in motivating individuals to seek and obtain support. These findings underscore the importance of screening for a range of mental health difficulties when assessing bereavement needs in primary care and community settings. Moreover, the consistent association between psychological distress and self-identified support needs suggests that enquiring about a person’s desire for support may be a simple and effective mechanism through which clinicians can identify individuals who would benefit from further psychological assessment or targeted bereavement interventions.

Inconsistent with prior research indicating that only 8% to 10% of bereaved individuals do not want support [17], this study found that nearly two-thirds did not want support. One possible explanation is the rather vague nature of the bereavement support question may have influenced these findings. Specifically, there are multiple different types of support that can occur after a loss including emotional support, instrumental support, and appraisal support [e.g., 26, 27], and the current study did not distinguish among them. Consequently, participants may have been responded to this question considering one specific form of support (e.g., support in coping with the emotional ramifications of the loss) or with several types simultaneously. Alternatively, the high proportion of participants not wanting support may reflect limited awareness of bereavement support in the general population. Research suggests that some bereaved individuals perceive formal support as being synonymous with talking therapies and is more appropriate for those who are considered to be “falling apart” or lacking nearby relatives [28]. In addition, bereaved individuals may be disincentivised from wanting support because of the frequent disconnect between the support offered and the support desired [15], as well as psychological (e.g.,. self-efficacy, perception, self-determination) and individual factors (e.g., ego, shame, introversion) that influence receptivity to support [29]. Overall, findings demonstrate that a significant proportion of bereaved individuals do not want support, and further research is required to examine different forms of support in greater detail and to understand the reasons underlying this reluctance.

Surprisingly, a considerable proportion of those who indicated that they did not want support reported receiving support regardless. Numerous studies have demonstrated that most individuals show resilience in the face of bereavement [30]. Although this resilience is widely recognized as reflecting better coping skills and adjustment to loss [31], many grief ‘myths’ persist, including beliefs about how people should respond to the death of a loved one and what constitutes an appropriate level of distress [32]. Consequently, when bereaved individuals do not conform to others’ expectations of grieving, people may feel more inclined to offer support, concerned that the person is not fully adjusting to the loss. This might explain why the proportion of the sample receiving support exceeds the proportion wanting support. Further investigation is necessary to ascertain whether this finding is replicated in different cultural settings, particularly considering that cultural norms around “normative” grieving vary greatly [33]. Likewise, due to the limited research in this area, future studies should explore cultural expectations and practices related to support following bereavement, including during the acute grief phase.

Consistent with earlier studies [18, 19], family and friends emerged as the most common and helpful sources of support. Likewise, support from fellow bereaved individuals was frequently received and perceived as beneficial, demonstrating the benefits of shared bereavement experiences [15]. Notably, the source of support deemed most unhelpful was general practitioners (GPs). Prior research has highlighted that GPs often feel ill-equipped and unprepared to provide bereavement support [34]. Since GPs are often the first point of contact for bereaved individuals, it is crucial to ensure they receive ongoing professional development in bereavement care [35]. Findings also showed how bereavement support services and grief counselling were some of the least commonly used sources of support. This aligns with research suggesting that only a small proportion of bereaved individuals experience pathological grief reactions [e.g., 21]. Moreover, studies have revealed that bereaved people tend to associate the support of grief services with the psychosocial aspects of grieving rather than the practical bureaucratic, legal, financial, and organisational aspects, which can be highly burdensome for the bereaved [28]. For instance, bereaved individuals may struggle with arranging funerals or developing new skills or roles previously assumed by the bereaved, and thus, these aspects of the bereavement experience should receive greater focus in bereavement care [28]. Although it was not possible to determine why grieving individuals used grief support services less frequently than, say, friends and family, investigating the types of support that these different sources provide is imperative to determine what kinds of support are considered most helpful. Finally, findings demonstrated an association between receiving support from more sources and poorer mental health. This likely suggests that those experiencing the highest levels of psychological distress following bereavement are more likely to be offered, and receive, support from a range of different (formal and informal) sources.

A strength of this study is the assessment of the types of support sources that are wanted and used by bereaved people, and their associations with loss-related characteristics and mental health. However, it was not possible to ascertain how participants interpreted support in the present study. As previously highlighted, bereaved individuals interpret support in various ways, including emotional support, instrumental support, or appraisal support [15]. Thus, future research should explore these different forms of support when investigating this topic. Similarly, a myriad of factors are likely to influence an individual’s perception of helpfulness of bereavement support, including the degree of alignment of the support with the needs of the bereaved individual [19], provider and recipient attributes (e.g., quality of the interaction, strength of relationship between recipient and provider) [27] the bereaved perceptions of what constitutes support (e.g., instrumental, emotional, instrumental) [15], and broader sociodemographic or mental health factors that can shape perceptions of support [36]. Similarly, there is likely variation in helpfulness within the different sources of support and additional sources of support that the present study did not capture. Moreover, examining the association between bereavement support and mental health outcomes in a cross-sectional study can be regarded as problematic in some respects. Specifically, it is not possible to determine whether the mental health problems preceded or succeeded the bereavement, particularly for those bereaved long-term. Future research should prioritize examining the association between bereavement support needs and pathological grief reactions longitudinally. Finallyd, the lack of information regarding indigenous or ethnic minority groups is a notable limitation, particularly given that prior research has shown how such groups are less likely to seek help for mental health [37]. Future research should pay careful attention to examining support seeking among these groups.

This study advances understanding of bereavement support by demonstrating that most individuals who seek support after a loss are able to access it and generally perceive it as beneficial. Notably more people receive support than actively desire it. The strong association between the desire for support and psychological distress, highlights the important of prioritising formal support for those experiencing elevated distress. Nonetheless, some bereaved individuals may exhibit symptoms of psychological distress following a bereavement that do not warrant formal support. Consequently, bereavement care should balance the reinforcement of natural social networks with the capacity to identify and respond to emerging distress. An “assets-based” [35], emphasizing the strength of existing community resources, may therefore represent an effective and efficient strategy for enhancing the efficiency and reach of bereavement care. Alternatively, some individuals who do not seek or want support may be experiencing symptoms that inherently reduce their motivation or capacity to do so, making them important targets for intervention. For instance, social withdrawal is a typical feature of depression [38] which commonly follows bereavement, while prolonged grief symptoms can heighten vulnerability to social loneliness [39]. Such withdrawal or loneliness can, in turn, increase risk of a plethora of maladaptive outcomes among bereaved individuals, including suicidality [e.g., 40], underscoring the importance of identifying and reaching those who express no desire for support, even through informal means.

## Data Availability

Neither the data nor the materials have been made available on a permanent third-party archive; requests for the data or materials should be sent via email to authors (m.shevlin@ulster.ac.uk or Philip.Hyland@mu.ie).
